# Elements in the Canine Distemper Virus M 3′ UTR Contribute to Control of Replication Efficiency and Virulence

**DOI:** 10.1371/journal.pone.0031561

**Published:** 2012-02-13

**Authors:** Danielle E. Anderson, Alexandre Castan, Martin Bisaillon, Veronika von Messling

**Affiliations:** 1 INRS-Institut Armand-Frappier, University of Quebec, Laval, Quebec, Canada; 2 Emerging Infectious Diseases Program, Duke-NUS Graduate Medical School, Singapore, Singapore; 3 University of Sherbrooke, Sherbrooke, Quebec, Canada; Virginia Polytechnic Institute and State University, United States of America

## Abstract

*Canine distemper virus* (CDV) is a negative-sense, single-stranded RNA virus within the genus *Morbillivirus* and the family *Paramyxoviridae*. The *Morbillivirus* genome is composed of six transcriptional units that are separated by untranslated regions (UTRs), which are relatively uniform in length, with the exception of the UTR between the matrix (M) and fusion (F) genes. This UTR is at least three times longer and in the case of CDV also highly variable. Exchange of the M-F region between different CDV strains did not affect virulence or disease phenotype, demonstrating that this region is functionally interchangeable. Viruses carrying the deletions in the M 3′ UTR replicated more efficiently, which correlated with a reduction of virulence, suggesting that overall length as well as specific sequence motifs distributed throughout the region contribute to virulence.

## Introduction

*Canine distemper virus* (CDV) belongs to the genus *Morbillivirus* within the family *Paramyxoviridae*. Like all other members of the order *Mononegavirales*, morbilliviruses are negative-sense, single-stranded RNA viruses. The morbillivirus genome is composed of six non-overlapping transcriptional units that are separated by untranslated regions (UTRs). The 3′ and 5′ UTRs are further separated by an intergenic triplet and contain transcriptional and translational initiation and termination signals [1], [2]. Genes are transcribed by a viral RNA-dependent RNA polymerase, which, using a mechanism known as the stop-start model of transcription [3], pauses at gene junctions and transcription can then either terminate or continue, creating a transcriptional gradient that is maintained throughout infection [4].

In morbilliviruses, the region between the matrix (M) gene stop codon and the fusion protein (F) signal peptide (Fsp) cleavage site, which separates the signal peptide from the F2 subunit of the mature F protein, is up to five times longer than the UTRs between all the other genes [5]. This increased length is due to a longer M 3′ UTR and, depending on the respective virus, either an equally long F 5′ UTR, or a combination of a shorter F 5′ UTR and an unusually long Fsp. While the F 5′ region of *Measles virus* (MeV) falls into the first group [6], the CDV F 5′ UTR is average in length, but the Fsp is extended [7]. When expressed outside the viral context, both CDV and MeV F 5′regions modulate the site of F protein translation initiation [6], [8], and may even act similarly to an internal ribosomal entry site [9]. In the viral context, deletion of the MeV or CDV F 5′ region alone resulted in increased F protein production and syncytia formation [7], [10], but did not affect replication efficacy or virulence in a human thymus/liver graft mouse model or ferrets, respectively [11], [12]. In contrast, replacement of the entire CDV M-F region with the regular UTR between the nucleocapsid (N) and phosphoprotein (P) genes resulted in attenuation and survival of all infected animals, indicating that the two parts act synergistically [11].

Less is known about the function of the long M 3′ UTR. In MeV, deletion of this region resulted in decreased M protein production, possibly due to loss of mRNA stability, and reduced replication [10]. In CDV, the entire M-F region is genetically variable among different strains [5], [14], and the M 3′UTR contains a conserved putative short open reading frame, but does not produce any polypeptides [13]. To further characterize the role of this region in pathogenesis, we first examined the strain specificity of the highly variable M-F region. To investigate the effect of the genetic variability, we initially produced recombinant viruses with exchanged M-F regions and evaluated their pathogenesis in ferrets. To determine the impact of M 3′ UTR length, a second set of recombinant viruses was generated and their growth characteristics and gene and protein expression levels were evaluated, and their virulence was assessed in ferrets.

## Results

### The variable M-F region is uniquely adapted to the morbillivirus species, but interchangeable within the genera

**Figure 1:**
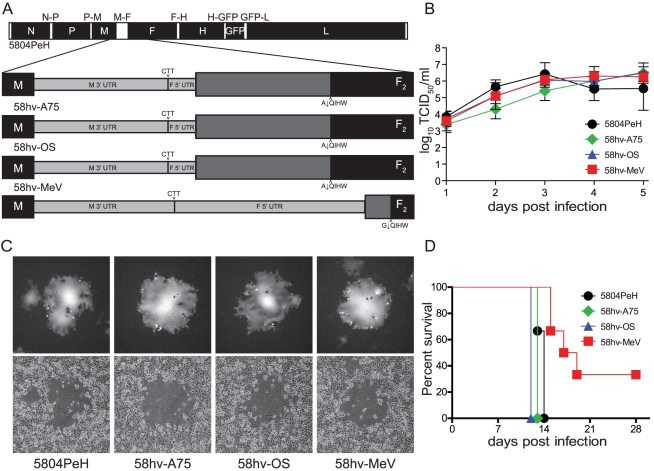
Growth characterization of recombinant M-F region exchange mutants. (**A**) Schematic drawing of the highly variable region exchanged in recombinant viruses. The recombinant 5804PeH genome is drawn to scale. Black and white boxes represent open reading frames and UTRs, respectively. The genes are indicated by their respective abbreviation. (**B**) Cell-associated growth curves of the parental and two recombinant viruses in VerodogSLAMtag cells. Cells were infected with a multiplicity of infection (MOI) of 0.01, and samples were harvested daily for five days. Titers are expressed as 50% tissue culture infectious doses (TCID_50_). Error bars indicate the standard deviation. (**C**) Syncytia formation in VerodogSLAMtag cells. Cells were infected with a MOI of 0.01 and overlaid with 0.5% methylcellulose. Photographs show a representative syncytia of one replicate taken 48 h post-infection using fluorescence excitation (top panels) and phase contrast (bottom panels) at 100× magnification. (**D**) Survival curve of ferrets infected with the different mutants. Animals were infected with 10^5^ TCID_50_ intranasally. Death of an infected animal is indicated by a step down on the graph.

The region between the M gene stop codon and the F sp cleavage site, while conserved in length, is highly variable [14], with differences between the two CDV wild type strains in this study, 5804P and A75/17, reaching 8% and between 5804P and the vaccine strain Onderstepoort (OS) 18%. This variability is 2–3 times higher than the nucleotide differences observed for either the M or mature F genes. Both wild type strains are lethal for ferrets. However, 5804PeH is not neuroinvasive with a disease duration of approximately 2 weeks, while A75/17 infected animals survive 3–5 weeks and virus is usually found in the central nervous system [15]. In contrast, the OS vaccine strain is completely attenuated and animals do not develop any clinical signs. To investigate the impact of this variability on pathogenesis, two recombinant viruses were produced: 58hv-A75, in which the variable M-F region of 5804PeH was replaced with that of A75/17 [16], another lethal CDV strain, and 58hv-OS, which carries the M-F variable region of the vaccine strain, Onderstepoort [17] (Fig. 1A). A third virus, 58hv-MeV, in which the M-F region of the MeV vaccine strain Moraten [18] replaces that of 5804PeH was generated to determine if the variable region could be exchanged between different morbillivirus species (Fig. 1A).

*In vitro*, all viruses reached the same peak titer of approximately 6.5×10^6^ TCID_50_/mL and displayed similar plaque morphology (Fig. 1B and 1C). To assess the virulence of the recombinant viruses, groups of three to six ferrets were infected intranasally with 10^5^ TCID_50_ of the different viruses. The exchange of the 5804PeH variable region with that from other CDV strains did not affect the disease course or outcome (Fig. 1D). All animals developed fever and rash 6–8 days after infection and ultimately succumbed to the disease within two weeks. In contrast, animals inoculated with 58hv-MeV experienced a prolonged disease and two out of six animals survived the infection (Fig. 1D). Both of the surviving animals developed fever and rash at day 10 post-infection. The rash cleared by day 14 post-infection in one animal, but remained until day 21 post-infection in the other animal. Thus, the M-F region is interchangeable among morbilliviruses without adversely affecting viral replication. However, exchange of the variable region outside the virus species attenuates the disease.

### The proximal half of the M 3′ UTR regulates replication efficiency and syncytia formation of the recombinant viruses

**Figure 2:**
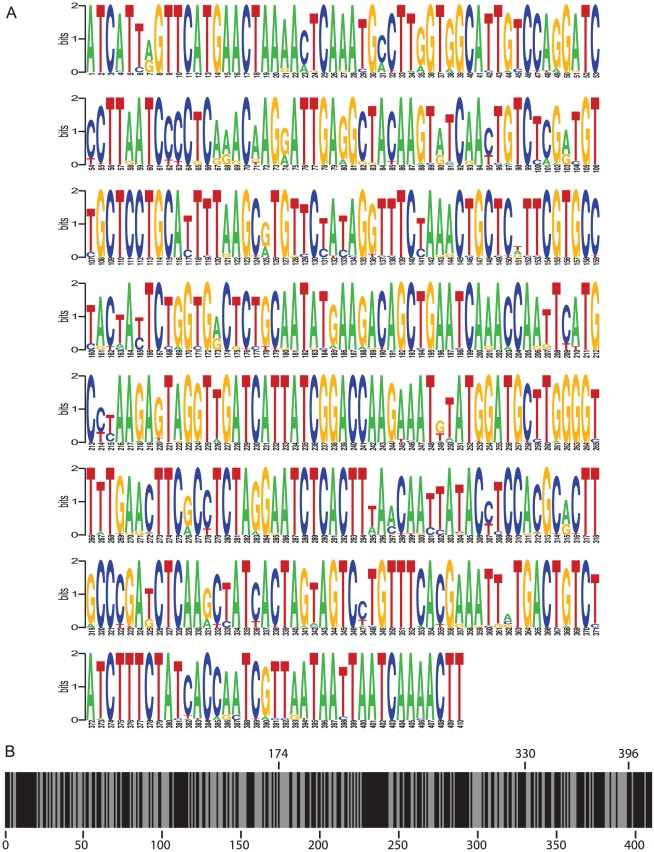
Conserved motifs within the M 3′ UTR. (**A**) A pictogram, assembled as described in Materials and Methods, shows the sequence and frequency of the 410 nucleotides of the M 3′ UTR. A base height of 2 bits indicates 100% conservation of that nucleotide across the 26 CDV sequences. The numbering below the pictogram indicates the position in the UTR, with 1 being first nucleotide following the M stop codon and positions 408–410 being the intergenic triplet. (**B**) Conserved regions within the M 3′ UTR. The M 3′ UTR consists of 410 nucleotides and each is represented by a line. Black lines indicate 100% conservation across the 26 CDV sequences and grey lines represent variable nucleotides.

We have previously shown that the long M-F UTR modulates CDV virulence through transcriptional control of F gene expression, and that this effect was primarily mediated by the M 3′ UTR [11]. To characterize this region in more detail, all 26 CDV sequences containing the complete M 3′ UTR were analyzed in an RNA frequency plot (Fig. 2A). Despite an overall variability of up to 18% between the vaccine and recent wild type isolates, multiple small blocks of conserved nucleotides were observed among the 410 residues between the M stop codon and the end of the M-F intergenic triplet (Fig. 2B). Based on this absence of larger conserved regions and the lack of any predictable secondary structure (data not shown), a partial deletion approach was chosen for further characterization of the M 3′ UTR.

**Figure 3:**
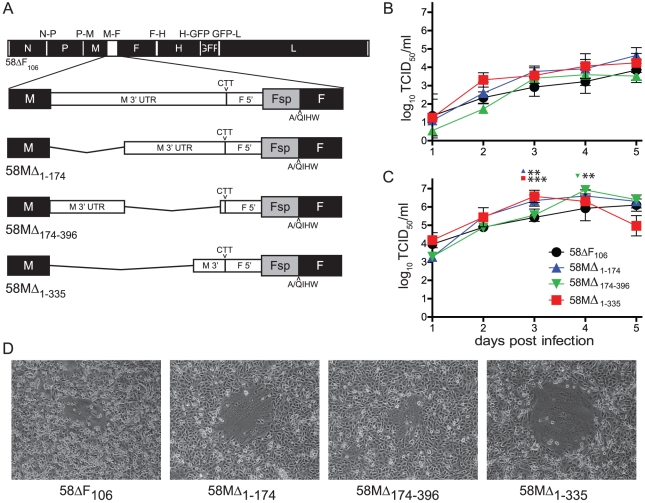
Generation and characterization of recombinant CDVs with alterations in the M 3′ UTR. (**A**) Schematic drawing of recombinant viruses produced. The recombinant 58ΔF_106_ genome is drawn to scale. Black and white boxes represent open reading frames and UTRs, respectively. The genes are indicated by their respective abbreviation. The M-F region of the recombinant viruses is expanded below. The UTRs are indicated by a thinner box, and the signal peptide coding region of the F gene (Fsp) is shaded. (**B** and **C**) Growth curves of the parental 58ΔF_106_ and the three recombinant viruses in VerodogSLAMtag cells expressed as titers of released (**B**) and cell-associated virus (**C**). Cells were infected with an MOI of 0.01, and samples were harvested daily for five days. Titers are expressed as TCID_50_ and error bars indicate the standard deviation. **, P<0.01, and ***, P<0.001. (**D**) Syncytia formation in VerodogSLAMtag cells. Cells were infected with a MOI of 0.01 and overlaid with 0.5% methylcellulose. Photographs show a representative syncytia of one replicate taken 48 h post infection using phase contrast at 100× magnification.

To assess the impact of the observed differences in downstream gene expression in the viral context, the respective deletions were introduced in the 58ΔF_106_ backbone [11]. This genome lacks the first 106 amino acids of the Fsp region, thereby assuring that all the effects observed are attributable to the M 3′ UTR. A total of three recombinant viruses were produced; 58MΔ_1–174_, which lacks the 174 nucleotides immediately downstream of the M gene stop codon, 58MΔ_174–396_, which lacks the following 222 nucleotides up to the conserved gene end sequence comprised of 11 nucleotides serving as polyA tail template, and 58MΔ_1–335_, which retains the last 61 nucleotides, thus reflecting a 3′UTR of typical length (Fig. 3A). The ‘rule of six’ [19] was respected during the creation of the full length constructs.

In VerodogSLAMtag cells, all viruses were released with an efficiency similar to the parental 58ΔF_106_ (Fig. 3B), while their cell-associated titers were up to 1 log higher, with 58MΔ_1–335_ and 58MΔ_1–174_ reaching significantly (p<0. 01) higher titers (Fig. 3C). Similar plaque morphology was observed for 58ΔF_106_ and 58MΔ_174–396_ (Fig. 3D), while syncytia produced by the other two viruses were larger, correlating with the observed increase in cell-associated titers. The increased replication efficiency and syncytia size of the viruses lacking the first part of the M 3′ UTR sequence indicates that the specific sequence of this region contributes to its function in the viral context.

### The M 3′ UTR does not affect overall viral gene expression

**Figure 4:**
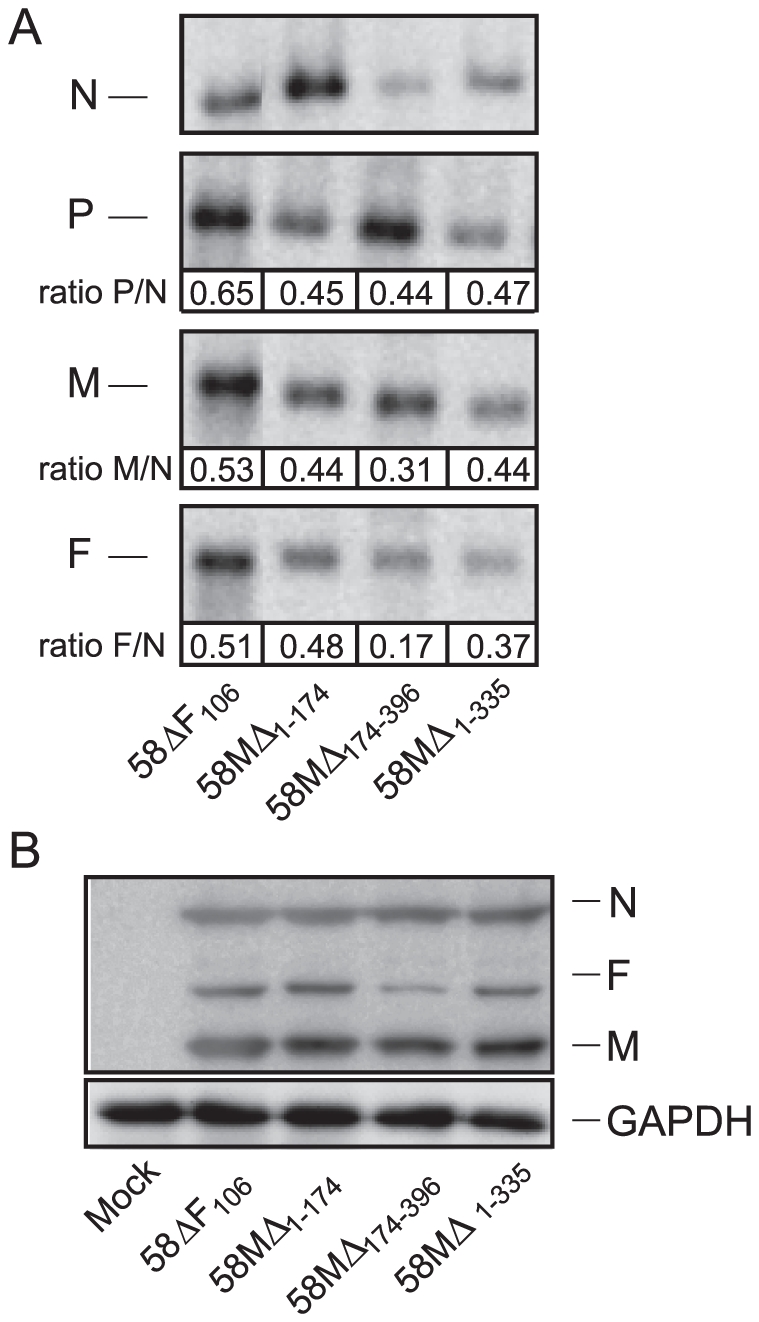
Effects of the M 3′ UTR on transcription and translation. (**A**) Northern blot analysis of viral mRNA with DNA probes. VerodogSLAMtag infected with the parental and recombinant viruses at an MOI of 1 were subjected to Northern blot analysis using DIG-labeled probes. Membranes were hybridized with DIG-labeled DNA probes for CDV N, P, M or F. Band intensities were determined by densitometry on non-saturated exposures using the Kodak Molecular Imaging software. The P, M and F expression were normalized by calculating the P/N, M/N and F/N ratios for each virus. The average mRNA ratios are shown beneath the appropriate bands and were calculated from at least four individual experiments. (**B**) Western blot analysis of viral N, M and F proteins. VerodogSLAMtag cells were infected with the parental and recombinant viruses at an MOI of 1 and overlaid with 0.5% methylcellulose. Cell lysates were extracted after 18 h and subjected to Western blot analysis. The membranes were probed with antibodies specific to the CDV N, M and F proteins. Intensities of N, M and F protein bands were determined by densitometry from non-saturated exposures using the Kodak Molecular Imaging Software. The M and F expression were normalized by calculating the M/N and F/N ratios for each virus and were calculated from at least four individual experiments.

Since we had previously observed that truncation of the F portion of the variable M-F region leads to an increase in F transcription and translation, we next examined whether the truncations in the M region would alter transcription of either upstream or downstream genes. Total RNA isolated from VerodogSLAMtag cells infected with a MOI of 1 was extracted and transferred onto nitrocellulose membranes. Membranes were hybridized with DIG-labeled DNA probes specific to N, P, M and F mRNAs (Fig. 4A). The mean P/N, M/N and F/N ratio of the parental 58ΔF_106_ and the other three recombinants was not significantly different. Western blot analysis of the corresponding proteins harvested 18 h after infection revealed some variability but no significant difference in M or F expression for any of the mutants compared to 58ΔF_106_ (Fig. 4B), indicating that alterations in the M 3′ UTR do not dramatically influence viral protein expression.

### The proximal part of the M 3′ UTR modulates early transcription and replication

**Figure 5:**
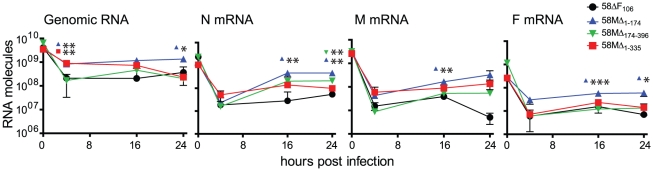
Effects of the M 3′ UTR on early transcription and replication. Quantitative real-time RT-PCR analysis of viral genome and N, M and F mRNA. VerodogSLAMtag cells were infected with the parental and recombinant viruses at a MOI of 1. RNA was extracted after 0, 4, 16 and 24 h and subjected to reverse transcription. Primer pairs specific to the CDV trailer, N, M and F genes were used. The number of RNA molecules in the sample was quantified by plotting the Ct values against standard curves. Error bars indicate the standard deviation. *, P<0.05, **, P<0.01, and ***, P<0.001.

To determine if the observed increase in replication efficiency of viruses lacking the first part of the M 3′UTR was due to subtle differences at early infection stages, we quantified viral genome and mRNA levels over the first 24 h after infection with a MOI of 1. Primers binding in the end of the L gene and in the trailer were used to detect genomic and antigenomic RNA, and primer pairs binding in the respective genes were used to measure mRNA levels. Even though the amount of genomic RNA in the inoculum was similar for all viruses, the levels for 58MΔ_1–174_ and 58MΔ_1–335_ were significantly higher (p<0.01) than the parental 58ΔF_106_ after 4 h (Fig. 5). Genome levels of 58MΔ_1–174_ remained significantly higher (p<0.05) throughout the experiment, indicating that this virus replicated faster immediately after infection.

The increased replication rate of 58MΔ_1–174_ correlated with a significantly higher production of viral mRNAs starting 16 h after infection (Fig. 5). Slightly higher mRNA levels, which were statistically significant in the case of 58MΔ_174–396_ N mRNA at the 24 h timepoint (p<0.01), were also observed for the other deletion mutants, albeit at a lesser degree. These results suggest that the proximal portion of the M 3′ UTR is involved in the control of genome replication initiation, while the entire region modulates viral gene transcription.

### Shortening of the M 3′ UTR extends CDV disease duration

**Figure 6:**
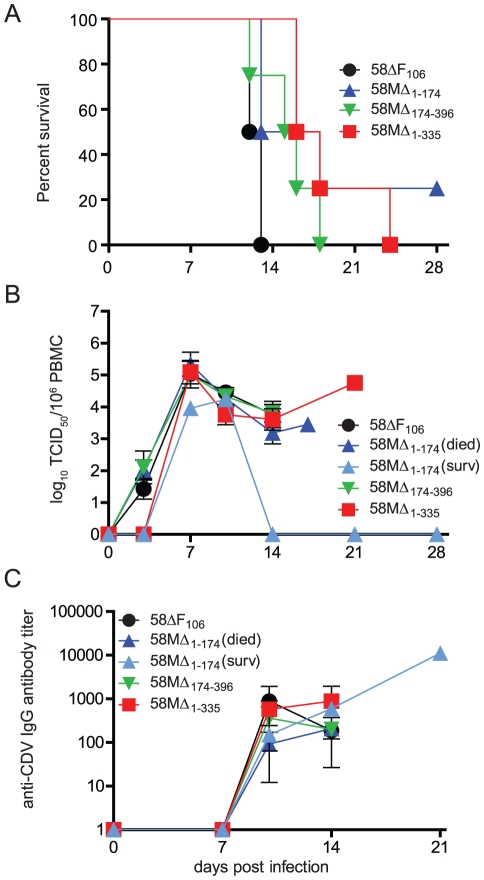
Characterization of M 3′ UTR mutants in ferrets. (**A**) Survival curve of ferrets infected with the different mutants. Animals were infected with 10^5^ TCID_50_ intranasally. Death of an infected animal is indicated by a step down on the graph. (**B**) Course of cell-associated viremia displayed as the number of CDV-infected cells per million peripheral blood mononuclear cells (PBMC). (**C**) CDV-specific IgG response in serum samples over the course of the disease. An immunoperoxidase monolayer assay was performed and antibody titers are displayed as reciprocals of the highest antibody dilution at which viral antigen was observed. Error bars indicate the standard deviation.

To determine the effect of M 3′ UTR alterations on pathogenesis, groups of four to six ferrets were infected intranasally with 10^5^ TCID_50_ of the different viruses. In contrast to 58ΔF_106_, which resulted in the death of all animals within two weeks, the disease course was prolonged for the majority of animals infected with the recombinant viruses. All animals developed fever and the characteristic rash 8–10 days after infection, but of those infected with 58MΔ_1–174_ only 50% succumbed to the disease within two weeks (Fig. 6A). One of the remaining animals died at 19 d.p.i., while the other recovered and survived the infection. Animals inoculated with 58MΔ_174–396_, which was the only virus to display a replication and syncytia phenotype similar to 58ΔF_106_ (Fig. 3), all died, but the disease course was slightly prolonged, with one animal surviving until 19 d.p.i. (Fig. 6A). The disease duration in animals inoculated with 58MΔ_1–335_ was prolonged even further, with one animal surviving until 24 d.p.i. (Fig. 6A).

The level of cell-associated viremia peaked in all groups at 7 d.p.i. (Fig. 6B), and survivors cleared the infection within three weeks after infection. However, 58MΔ_1–335_, which resulted in the longest disease, was only detected on day 7 after infection, indicating that its replication *in vivo* was slightly delayed. To monitor the CDV-specific antibody response, total antiviral IgG was quantified in serum samples from different time points (Fig. 6C). Animals that succumbed to the disease within two weeks were unable to mount a sustained CDV-specific response, while all survivors reached titers above 10,000 within three weeks (Fig. 6C). No sequence changes in the region between the start of the M gene and the end of the F gene were found in viruses isolated from survivors at day 14 or at the time of euthanasia. Altogether, the assessment of the recombinant viruses in ferrets demonstrates that shortening of the M 3′ UTR gradually reduces virulence, indicating that its overall length also contributes to its function.

## Discussion

The morbillivirus M-F region, comprising the M-F UTR and the F protein signal peptide, is considerably longer and more variable than all other UTRs, reaching up to 18% difference between CDV strains [5], [14]. While different regulatory functions of this region have been demonstrated for MeV and CDV [10], [11], a more detailed characterization has been challenging. In this study, we demonstrate that the highly variable M-F region is functionally interchangeable between CDV strains regardless of their virulence, indicating that the essential regulatory elements are conserved. Exchange of the highly variable region with MeV lead to partial attenuation, illustrating that these regulatory elements are similar but not conserved within the genus. We have previously shown that the regulatory function of this region resides mostly in the M 3′UTR, since shortening of the long signal peptide in the F 5′ region alone did not alter the *in vitro* phenotype or affect pathogenesis [11]. To further characterize the role of the M 3′UTR, two complementary partial and one complete deletion mutant were generated. While only the proximal part of the M 3′ UTR modulated the initiation of viral genome replication, overall shortening of the region correlated with prolonged disease duration in ferrets, indicating that specific sequence elements as well as general length are required to maintain wild type virulence.

### The genetic diversity of the CDV M-F variable region does not alter its function

In positive strand RNA viruses, the 5′ and 3′ UTRs form distinct secondary and tertiary structures, and genetic variability in these regions influences overall viability and pathogenesis [20]. It was thus conceivable that the observed genetic variability in the long CDV M-F region also contributes to strain-specific differences in pathogenesis and disease severity. The genomic RNA of negative strand RNA viruses is usually encapsidated by the viral nucleoprotein [21], making the formation of secondary or tertiary structures less likely, while the positive stranded mRNA transcripts could potentially form such structures. However, an *in silico* analysis of region did not reveal any distinct secondary structures in either the genomic or mRNA sequences, and its replacement with the corresponding region from different strains had no effect on disease phenotype, indicating that the genetic variability reflects an accumulation of random mutations at sites that are under no selective pressure. This conclusion is further supported by a recent study comparing MeV wild type and SSPE strains, which found the highest intra- and inter-genotype variability in the M-F region but was unable to identify changes that correlated with the disease phenotype [22].

### Length and sequence are important for M 3′UTR function

Previous investigations of the morbillivirus M-F region indicate that this region modulates up-and downstream gene expression, thereby influencing replication, syncytia phenotype, and virulence [10], [11]. Despite the considerable variability regarding the roles ascribed to the M 3′ and F 5′ part of the region, there is a consensus that the MeV F 5′UTR and the corresponding long CDV Fsp have an inhibitory effect on F protein expression [7], [23]. In contrast, the role of the M 3′ UTR is less clear. It was recently demonstrated that a putative open reading frame present in the M 3′ UTR of some CDV strains is not used [13], confirming that the function of this regions is primarily on the RNA level. In MeV, deletion of the M 3′UTR resulted in reduced M protein expression and delayed replication, even though the virus retained the parental syncytia phenotype [10], while no effect on up- or downstream gene expression was observed in a bi-fluorescent minigenome system [23]. We previously observed that replacement of the CDV M 3′ UTR with the N 3′ UTR led to increased downstream gene transcription in VerodogSLAM tag cells [11], but did not ascertain whether the differences in overall length or in the sequence were the determining factor. The increase in viral mRNA transcription observed in the same cells upon shortening of the M 3′ UTR confirms the importance of the length of this region, while the control of genome replication mediated by the proximal part points towards a contribution of the sequence itself. Hence, elements within the proximal M 3′ UTR contribute to the control of viral replication. How this regulation affects replication in different target cells is the subject of ongoing investigations.

### The entire M 3′ UTR contributes to the wild type disease phenotype

Despite its overall genetic variability, the CDV M 3′ UTR contains multiple blocks of completely conserved nucleotides. Here we observed that disease duration was inversely correlated with the length of the M 3′ UTR, with partial deletions extending survival by five days and the complete deletion by 10 days, indicating a certain level of redundancy and synergism among these elements. The disease duration of the mutant viruses is within the normal range of CDV wild type strains and similar to the neurovirulent A75/17 strain. While still lethal, this virus is less immunosuppressive [16], suggesting that the dysregulation of replication observed *in vitro* may facilitate the immune recognition of the 3′ UTR deletion mutants thereby extending survival. A similar effect has been observed in Borna disease virus, where instability elements in mRNAs, which are modulated by the presence or absence of neighboring sequences, have been suggested to facilitate persistent infection [24]. In addition, it is conceivable that the long M 3′UTR gives rise to small regulatory RNA elements that modulate transcription or viral replication as recently reported for influenza A virus [25].

In contrast to the N-P UTR replacement mutant which was completely attenuated [11], the complete M 3′UTR deletion mutant 58Δ_1–335_ remained lethal, even though the length of these UTRs was similar, demonstrating a contribution of gene-specific transcription start and end sequences to virulence. An impact of transcription start and end sequences on gene transcription efficiency has been reported for several paramyxoviruses [26], [27], but a direct role in pathogenesis has so far been speculative. While it remains unclear how the M 3′ UTR exerts its effects on genome replication and mRNA transcription, our results illustrate the importance of tight replication control for morbillivirus pathogenesis.

## Materials and Methods

### Ethics statement

All animal experiments were conducted in accordance with the guidelines of the Canadian Council of Animal Care and were approved by the Institutional Animal Care and Use Committee of the INRS-Institut Armand-Frappier (#0808-01).

### Cells and viruses

VerodogSLAMtag cells [28] and 293 cells (ATCC CRL-1573) were maintained in Dulbecco's modified Eagle's medium (DMEM, Invitrogen) with 5% fetal calf serum (FCS, Invitrogen). The parental recombinant CDV 5804PeH [29], A75/17 [16], Onderstepoort [17], and 58ΔF_106_ (2), and all recombinant viruses constructed in this study were propagated in VerodogSLAMtag cells.

### Generation of recombinant viruses

All recombinant viruses were generated in the pBR-5804PeH [29] background. Recombinant PCR fragments were produced by overlap extension PCR [30] and introduced into the 5804PeH genome using *Sac*II and *Bsr*GI. Three recombinant viruses carrying the variable region spanning from the M stop codon to the Fsp cleavage site of CDV A75/17 [16], CDV Onderstepoort [17] or MeV Moraten [18] were constructed, yielding 58hv-A75, 58hv-OS and 58hv-MeV, respectively. The pBR-58ΔF_106_ plasmid [11] was used for the construction of all deletion mutant viruses. As described above, recombinant PCR fragments were introduced via the *Sac*II and *Bsr*GI restriction sites, resulting in 58MΔ_1–174_, 58MΔ_174–396_ and 58MΔ_1–336_. The rule of six [19] was respected in all recombinant viruses and all insertions were verified by sequencing.

Recombinant viruses were recovered and characterized as previously described [11]. Briefly, 5×10^5^ 293 cells in 6-well plates were transfected with 4 µg of plasmids encoding the recombinant full-length CDV plasmid in combination with 0.5, 0.1, 0.5 and 0.7 µg of MeV N, P, polymerase (L) and T7 polymerase expression plasmids, respectively, using Lipofectamine 2000 (Invitrogen). Two days post-transfection, the 293 cells were co-cultured with 5×10^6^ VerodogSLAMtag cells and maintained in DMEM containing 5% FCS until syncytia were observed. Syncytia were then transferred onto fresh VerodogSLAMtag cells to produce virus stocks.

For virus kinetics, VerodogSLAMtag cells were infected at a multiplicity of infection (MOI) of 0.01 50% tissue culture infective dose (TCID_50_) and samples were harvested daily for 5 days following the infection, and the cell-associated and cell-free titers were determined by limited dilution. Photographs of representative syncytia were taken for each replicate using an Eclipse TE2000-U compound microscope with a DXM1200F digital camera (Nikon). Statistical analysis was performed using a one way ANOVA with Tukey's multiple comparison test.

### Animal experiments and assessment of virulence

The experiments were performed as described previously [28] using unvaccinated ferrets (*Mustela putoris furo*) 16 weeks and older (Marshall Farms). Groups of three to six animals were infected intranasally with 10^5^ TCID_50_ of each virus. Animals were observed daily, and body temperature and clinical signs were recorded. Blood samples were collected from the jugular vein under general anesthesia on days 3, 7, 10 and 14 post-infection and weekly thereafter. Cell-associated viremia was quantified by measuring the virus in the infected PBMCs as previously described [31]. RNA was isolated from the PBMCs collected on day 14 or the time of euthanasia, respectively, and the region encompassing the M gene, the M-F UTR, and the F gene was amplified and sequenced to assess the accumulation of mutations.

### RNA sequence and structure analysis

All available sequences of the CDV M 3′ UTR were extracted from the GenBank database (www.ncbi.nlm.nih.gov). A total of 26 sequences were obtained (Accession numbers: AY386316, AF164967, EU726268, AF378705, AY649446, AY542312, EU716337, AY443350, AY466011, AY445077, FJ694850, FJ694844, FJ694842, FJ694848, FJ694845, FJ694847, FJ694846, FJ694843, AF026243, AF026239, AF026242, AF026237, AF026232, AF026235, AF026240, AF026234). Sequences were exported in FASTA format and an RNA CLUSTAL W multiple sequence alignment was generated using the R-Coffee server [32]. WebLogo program (http://weblogo.berkeley.edu/) [33] was used to generate a frequency plot of the 410 bp of the M 3′ UTR. The secondary structures of lowest free energy were predicted using the MFOLD program [34].

### Northern blot analysis

The PCR DIG Probe Synthesis Kit (Roche) was used following manufacturer's instructions for the generation of N, P, M and F gene-specific digoxigenin (DIG)-labeled probes, using pBR-5804PeH as template. VerodogSLAMtag cells in 100 cm^2^ plates were infected with the different viruses at a MOI of 1, overlaid with media containing 0.5% methylcellulose (w/v) and incubated at 32°C for 18 h. Total RNA was isolated from infected cells using the RNeasy RNA extraction kit (Qiagen), and Northern blot assays were performed as previously described [11]. Membranes were exposed on a luminescent image analyzer (Kodak) and quantified using Molecular Imaging Software (Kodak).

### Polyacrylamide gel electrophoresis and Western blotting

VerodogSLAMtag cells in 6-well plates were infected with the different viruses at a MOI of 1, overlaid with media containing 0.5% methylcellulose (w/v) and incubated at 32°C for 18 h. Cells were washed with PBS, lysed in 200 µL of RIPA buffer (1 mM PMSF, 1% sodium deoxycholate, 50 mM Tris-HCl, pH 7.4, 1% Triton-X100, 0.1% SDS, 150 mM NaCl) on ice for 10 min, and centrifuged at 17,000 *g* for 10 min at 4°C. Cell lysates were separated by 10% SDS-polyacrylamide gel electrophoresis and transferred on Immobilon-P PVDF membrane (Millipore Corporation). Membranes were blocked with 5% skim milk in TBS-T (50 mM Tris-HCl, pH 7.4, 150 mM NaCl, 0.1% Tween 20), and incubated with CDV N and F protein-specific rabbit anti-peptide sera [11] and MeV M monoclonal antibody (MAB8910, Millipore), followed by horseradish peroxidase-conjugated secondary antibodies. Proteins were visualized using the ECL Plus Western Blotting Detection system (GE Healthcare), exposed on a luminescent image analyzer (Kodak), and quantified using Molecular Imaging Software (Kodak).

### Immunoperoxidase monolayer assay (IPMA)

The IPMA was performed as previously described [11], [35], with the exception of the infection of VerodogSLAMtag cells, which were infected with 58ΔF_106_. Titers were expressed as reciprocals of the highest antibody dilution at which intracellular viral antigen was detected by light microscopy.

### Quantitative RT-PCR

VerodogSLAMtag cells in 6-well plates were infected with the different viruses at a MOI of 1 and incubated at 32°C. Cells were harvested at 4, 16 and 24 h post-infection and total RNA was isolated from infected cells using the RNeasy RNA extraction kit (Qiagen). For the 0 h sample, RNA was isolated from virus inoculum equivalent to the amount used for infection. RNA for the quantitative RT-PCR standard curve was generated by *in vitro* transcription using the Riboprobe System-T7 kit (Promega), according to manufacturer's specifications. The RNA was quantified using the NanoDrop 2000 spectrophotometer (Thermo Scientific) and RNA integrity determined by agarose gel electrophoresis. The number of RNA molecules produced by *in vitro* transcription was calculated using the following formula: RNA molecules/µL = [µg RNA/transcript length×340]×[6.022×10^23^].

RNA samples were reverse transcribed using the Tetro cDNA Synthesis kit (Bioline) and random hexamers following the manufacturer's instructions. The quantitative PCR was performed using the QuantiFast SYBR Green PCR kit (Qiagen) and a Rotor-Gene Q apparatus (Qiagen). Each 10 µL PCR reaction contained 5 µL 2× QuantiFast SYBR Green PCR mastermix, 1 µL of each 10 mM primer, 1 µL cDNA template and 2 µL H_2_O. Every PCR was performed as follows: initial PCR activation at 95°C for 5 min and 45 amplification cycles consisting of a 95°C denaturation for 10 sec and a 60°C annealing/extension for 30 sec. Melting curve analysis was performed to confirm reaction specificity. Each sample was analyzed in triplicate and each experiment was carried out at least twice. Amplicons were quantified by plotting the Ct values against standard curves made using 10-fold dilutions of cDNA produced from *in vitro* transcription RNA samples.
